# Supplementary figures and images for: A critical analysis of UK media characterisations of Long Covid in children and young people
Source: PLOS Glob Public Health. 2024 Nov 27;4(11):e0003126. doi: 10.1371/journal.pgph.0003126 (PMC11602070; doi:10.1371/journal.pgph.0003126)

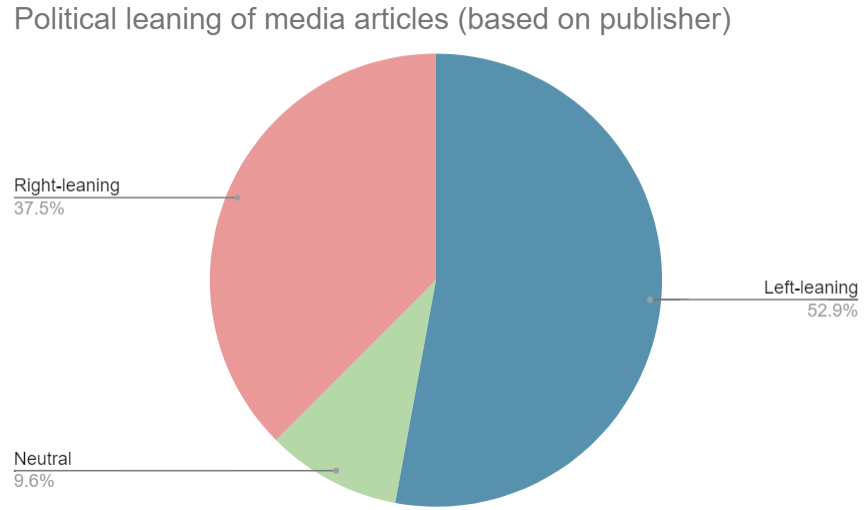

Supplement: S1 Fig — (TIF) [file pgph.0003126.s001.tif]

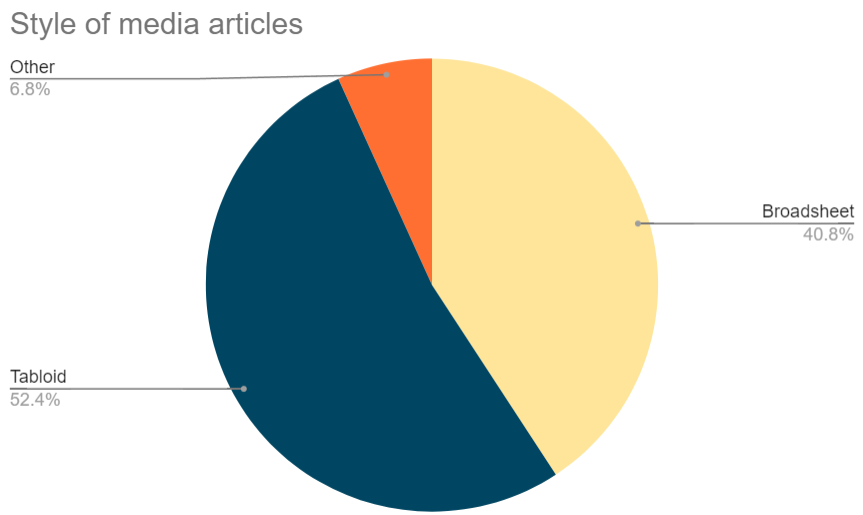

Supplement: S2 Fig — (TIF) [file pgph.0003126.s002.tif]

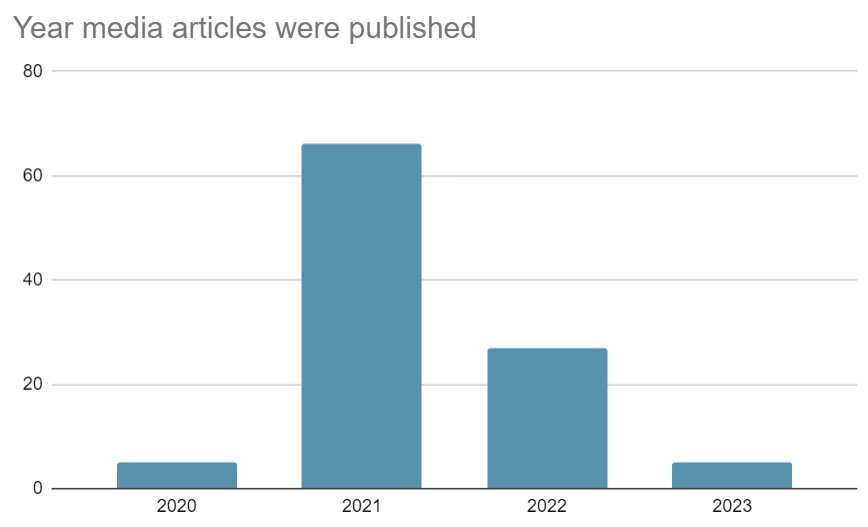

Supplement: S3 Fig — (TIF) [file pgph.0003126.s003.tif]

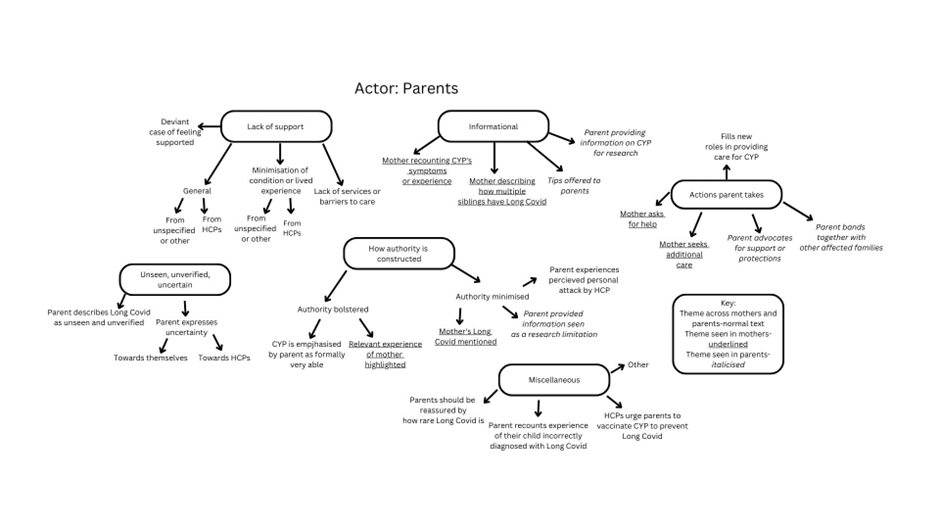

Supplement: S4 Fig — (TIF) [file pgph.0003126.s004.tif]

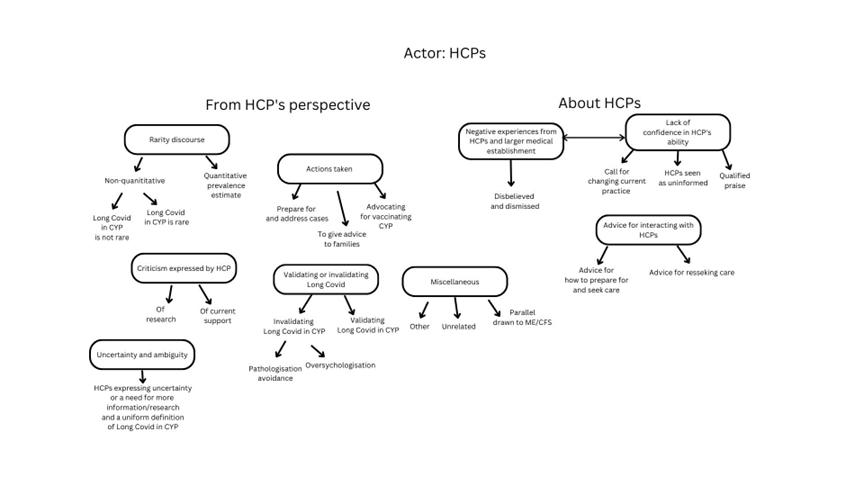

Supplement: S5 Fig — (TIF) [file pgph.0003126.s005.tif]

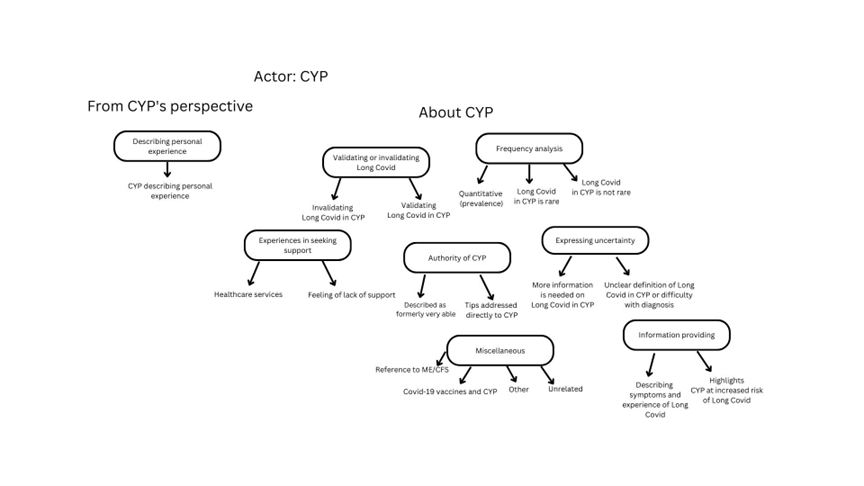

Supplement: S6 Fig — (TIF) [file pgph.0003126.s006.tif]
