# Supplementary material for: A critical analysis of UK media characterisations of Long Covid in children and young people
Source: PLOS Glob Public Health. 2024 Nov 27;4(11):e0003126. doi: 10.1371/journal.pgph.0003126 (PMC11602070; doi:10.1371/journal.pgph.0003126)
Supplement: S7 Table — (DOCX) [file pgph.0003126.s013.docx]

S7 Table. Trustworthiness of findings.

| **Credibility:** The primary coder/researcher (CC) has completed several courses on qualitative research and social science methodology as part of her completed master’s and bachelor’s degrees. In addition, the primary coder worked under the mentorship of several coauthors who have expertise in qualitative research and/or discourse analysis. SM, an experienced qualitative researcher, provided three designated research support sessions designed to improve the quality of the analyses. This was in addition to the guidance offered by the NA, MK, and DC and served as peer debriefing (at the time SM was not part of the research). In the sessions, SM functioned as the external researcher and discussed potential insights and biases with the primary coder.  CC, MK, and NA engaged in co-design with SM to build engagement with relevant actors. This enabled the researcher to triangulate and contextualise findings.  CC and DC located and highlighted deviant case examples to ensure that themes were not cherry picked or presented incompletely. DC reviewed all data that were assigned codes included in findings. |
| --- |
| **Transferability:** The researcher demonstrated the transferability of the findings through referencing parallels between Long Covid and ME/CFS. In addition, comprehensive accounts of the data set and methods for actor selection were given. This enables readers to assess the rigour and transferability of findings. |
| **Dependability:** Dependability was demonstrated through a detailed description of the methods and the inclusion of inductive thematic maps prior to conceptualisation of the data considering the framework of epistemic injustice. The methods were presented in chronological order alongside a rationale, which served as an audit trail of processes. |
| **Confirmability:** The researcher established confirmability through transparent reporting of the methods and analysis. Preliminary results were shared with coauthors and outside researchers with knowledge on Long Covid. Themes were finalized with input from coauthors. The inclusion of the inductive thematic maps demonstrate the full scope of the data beyond what was included in the results and discussion. In addition, the researcher also engaged in reflexivity (reflexivity statement available upon request). |
